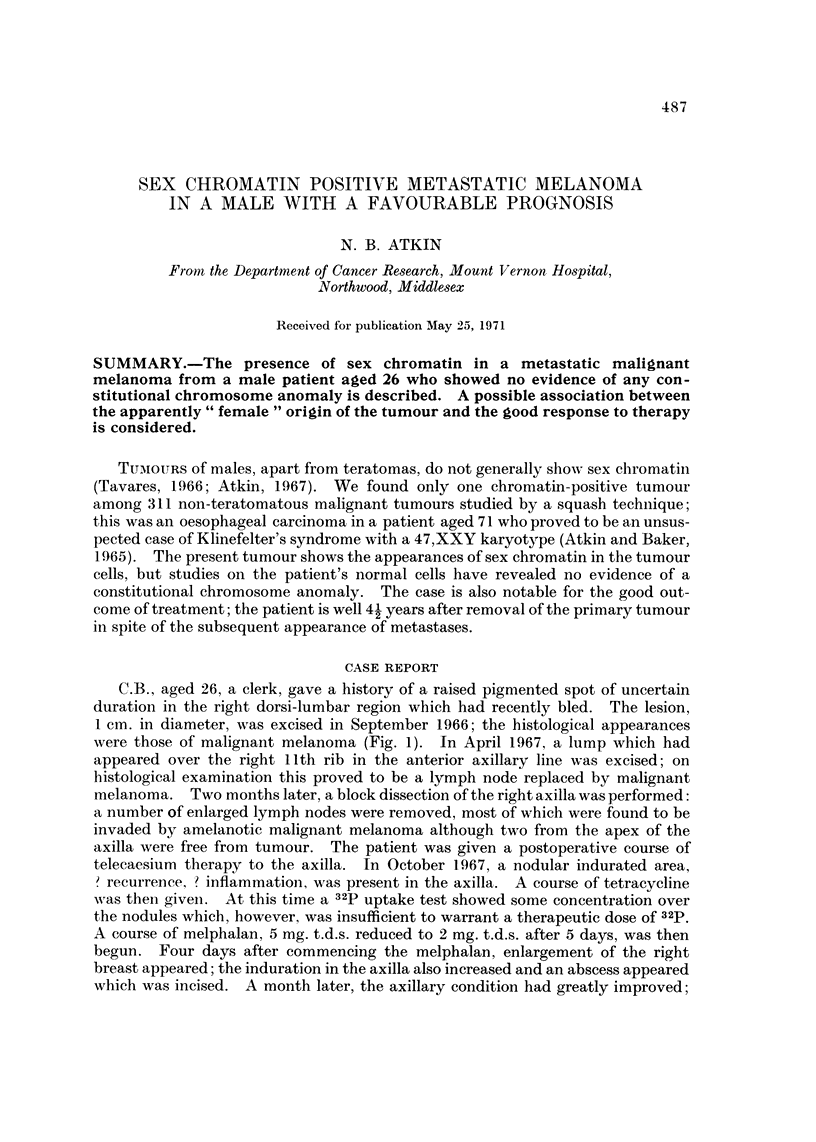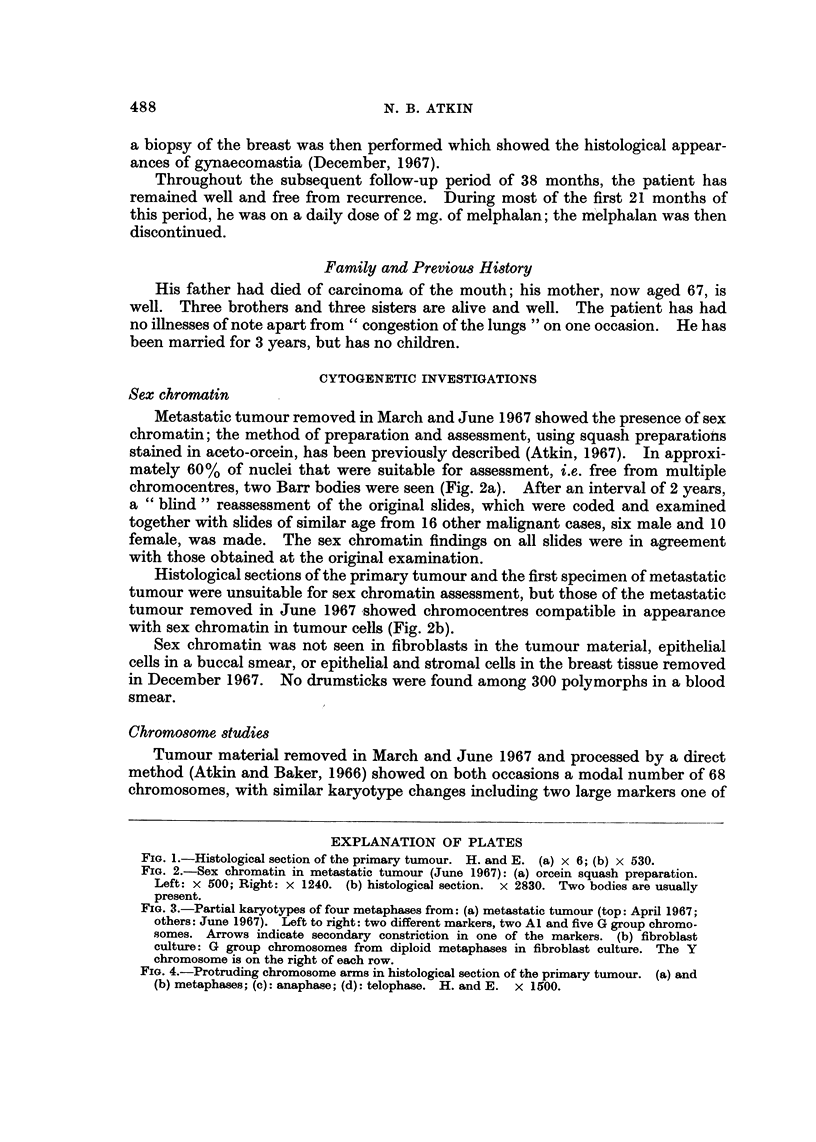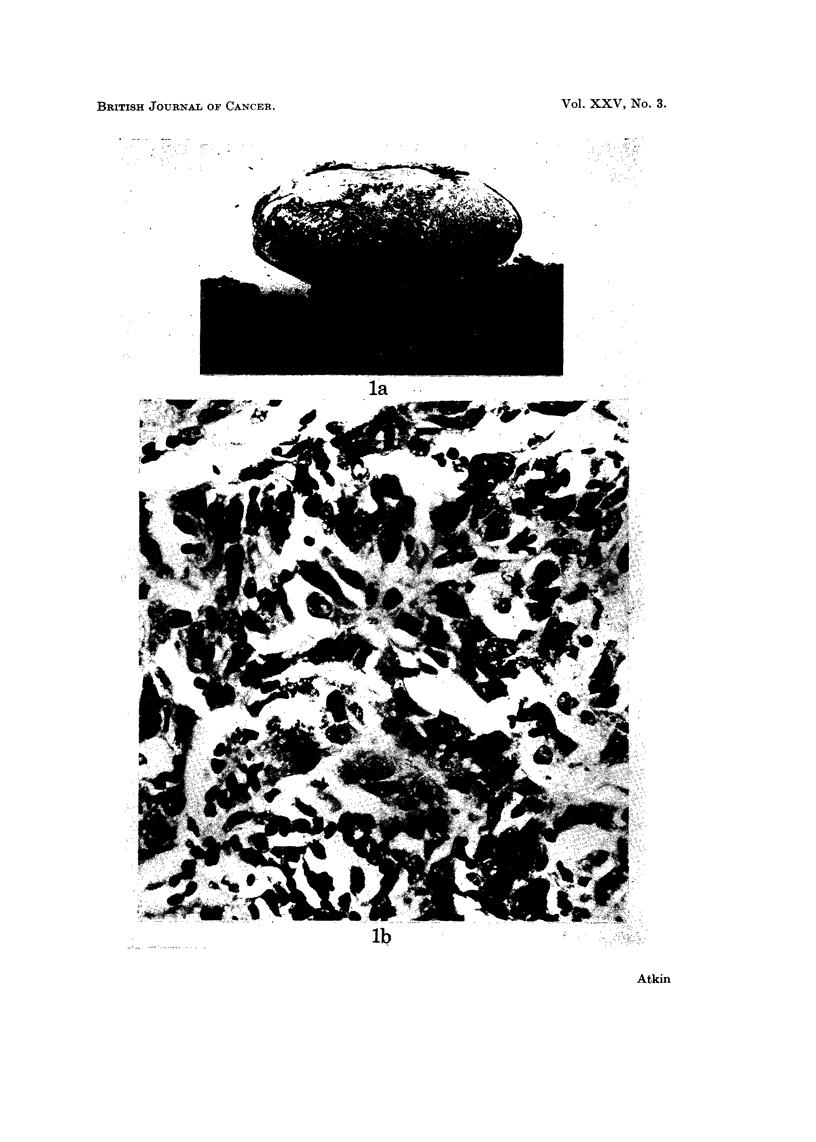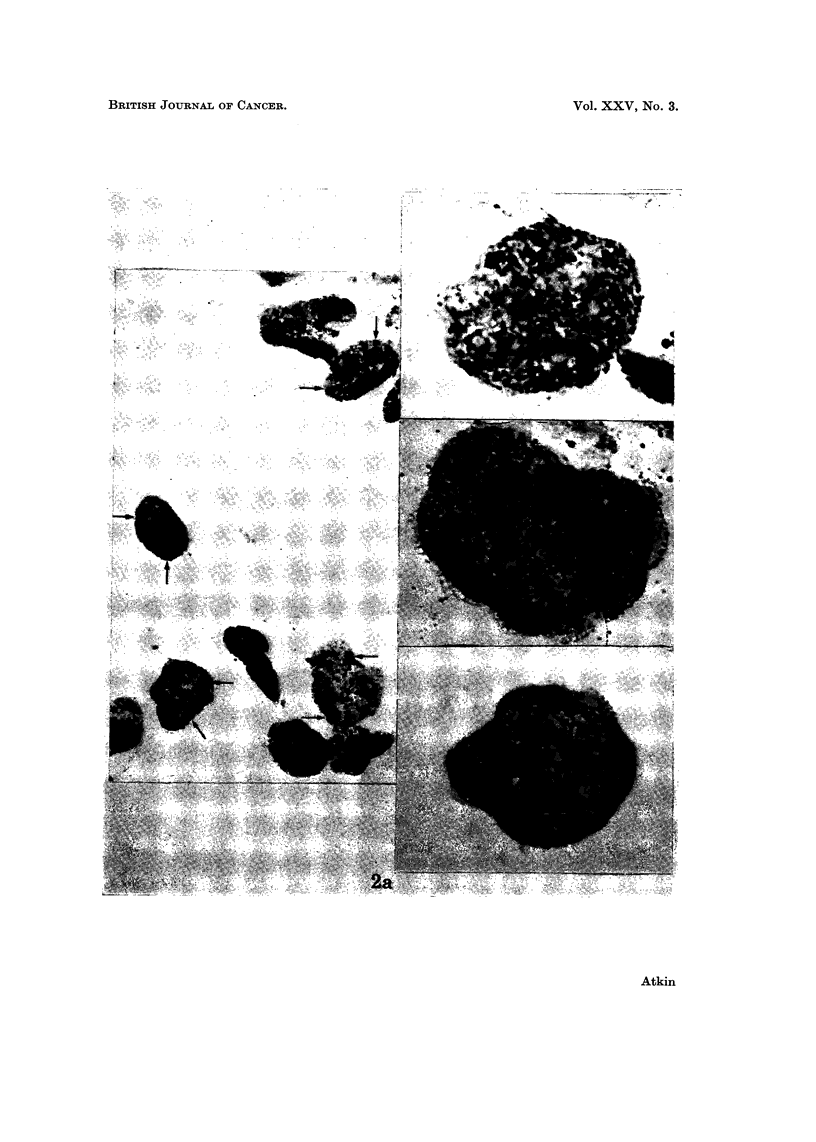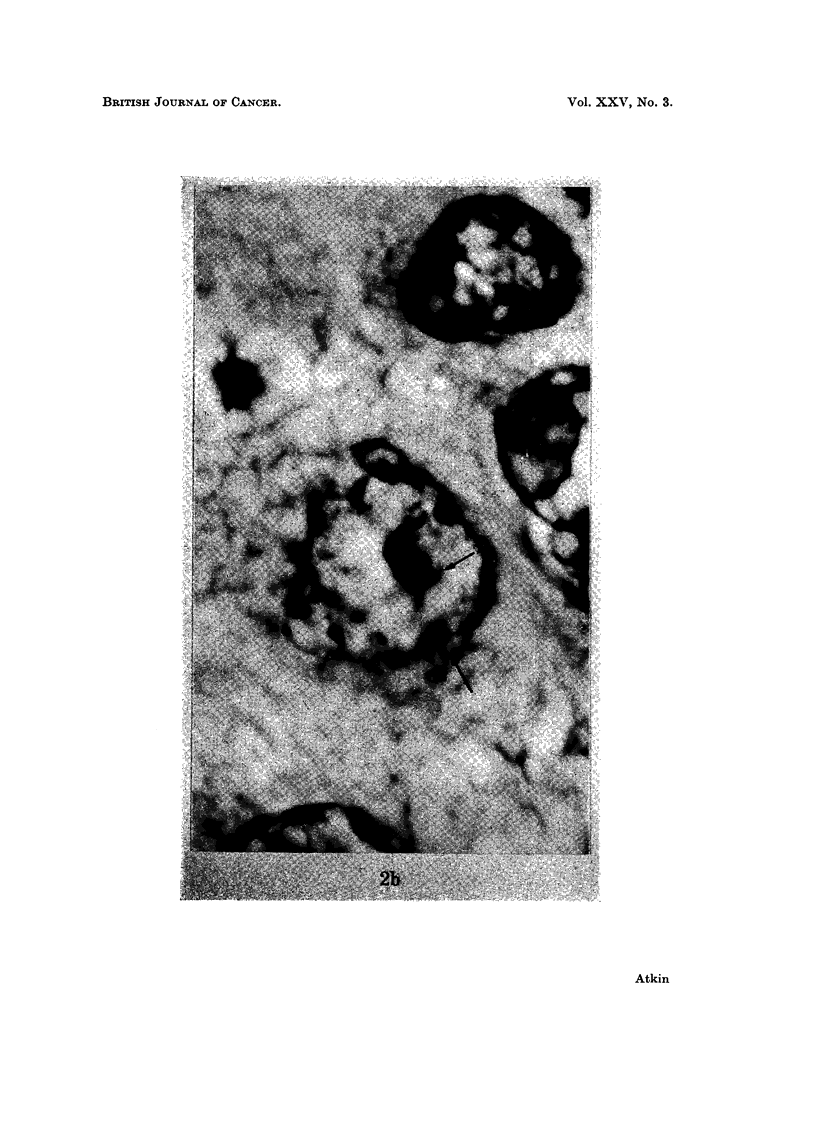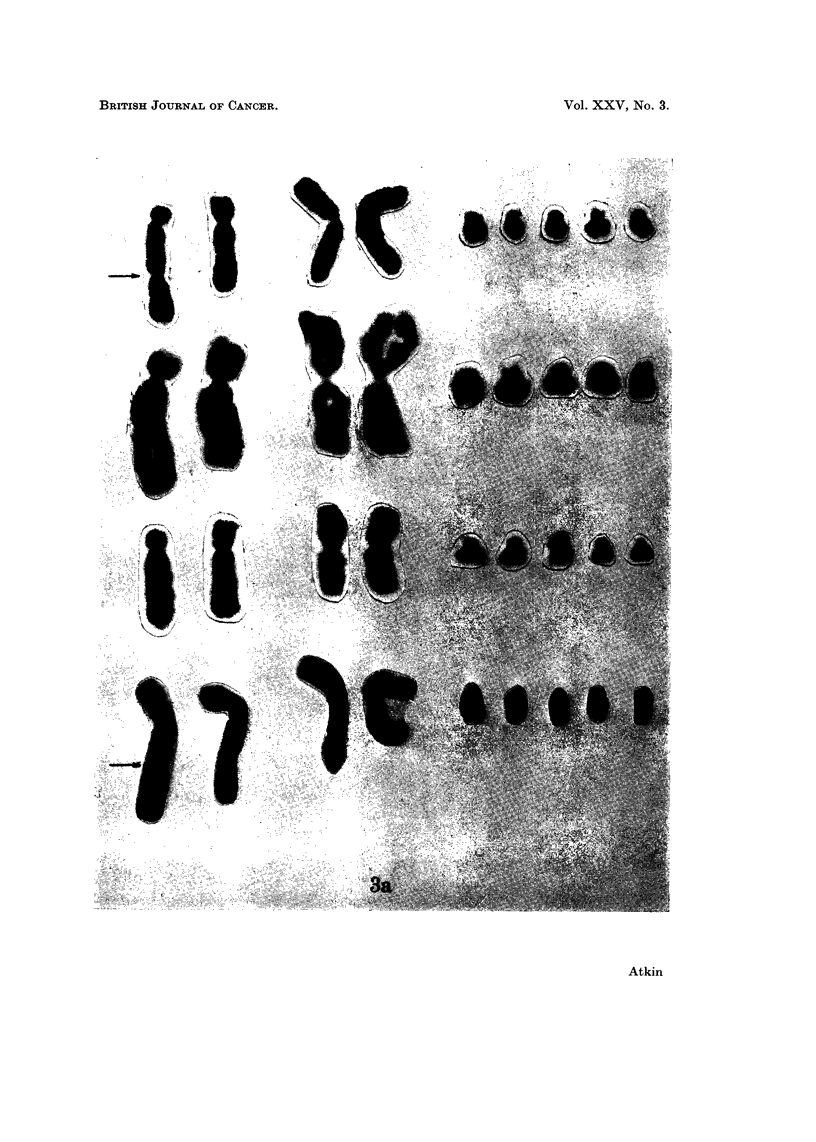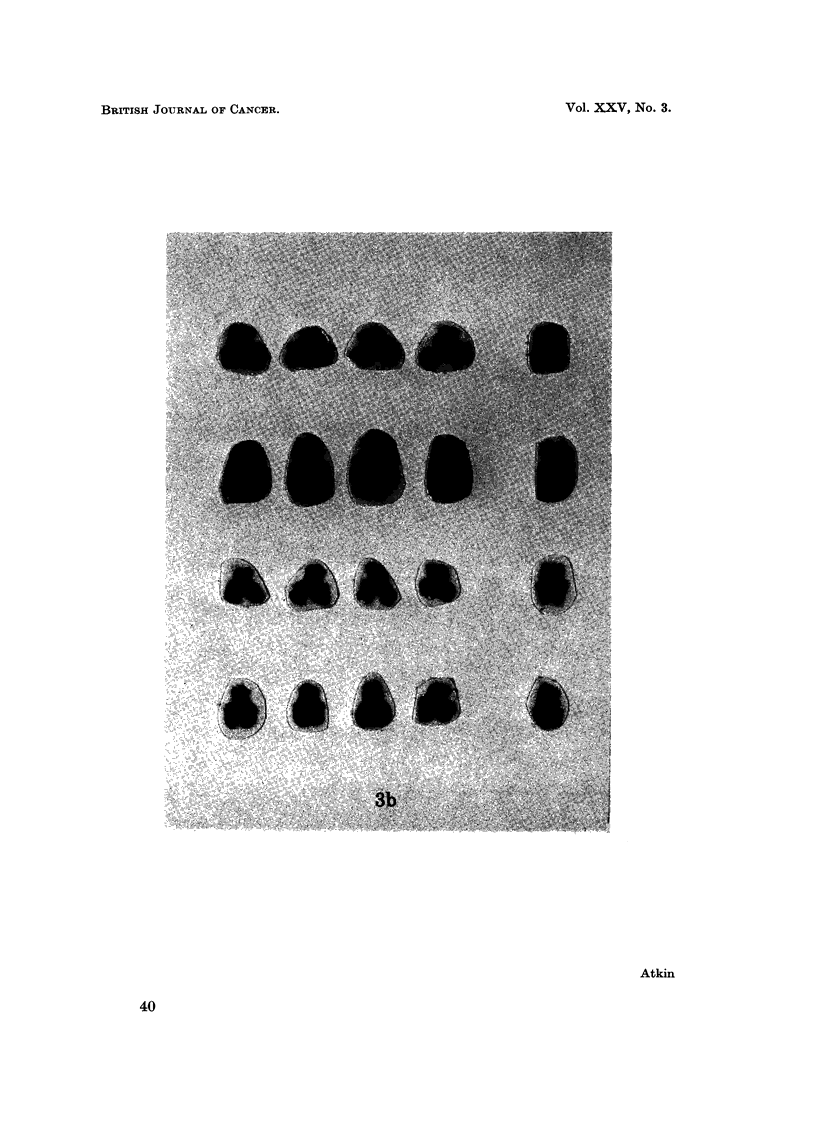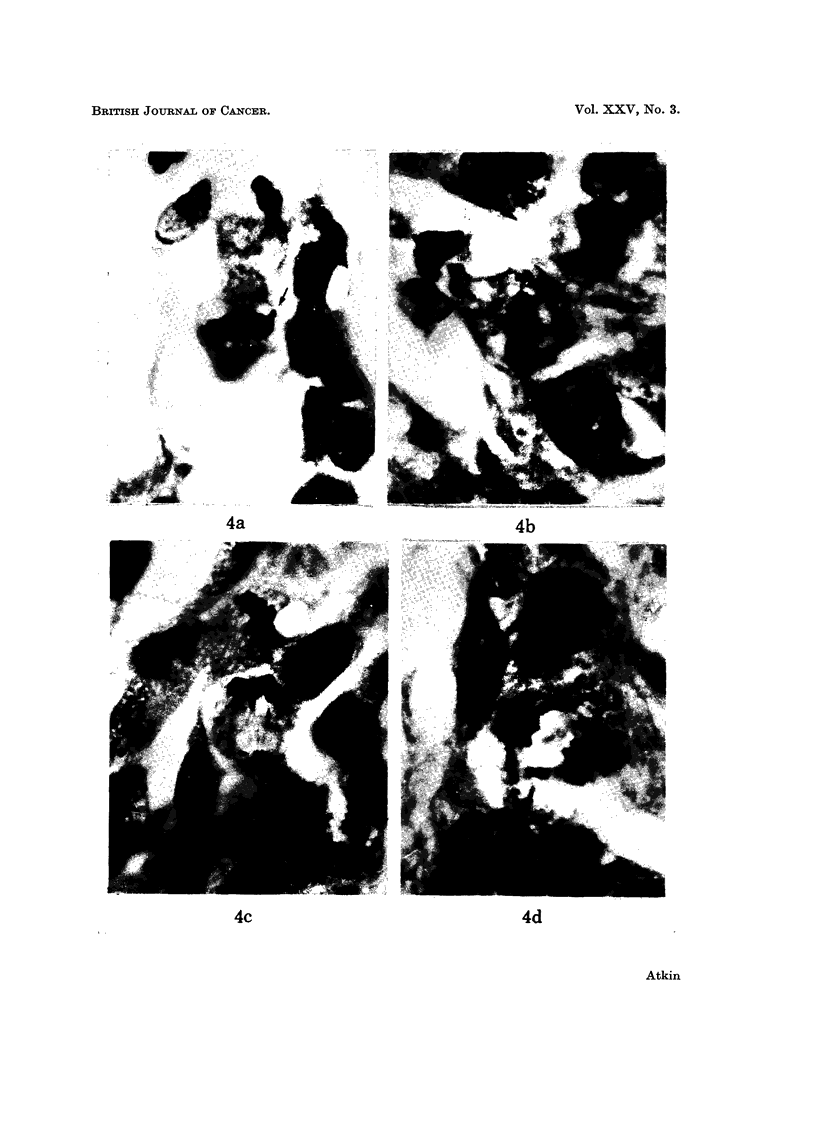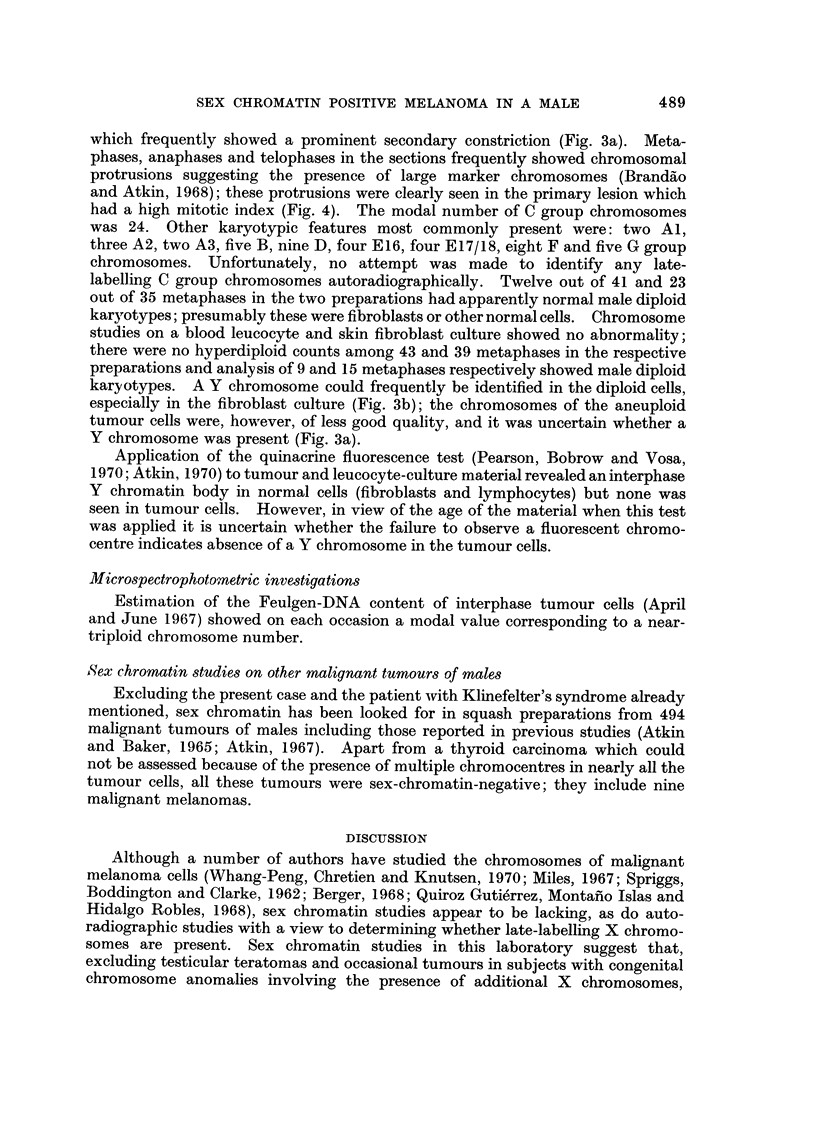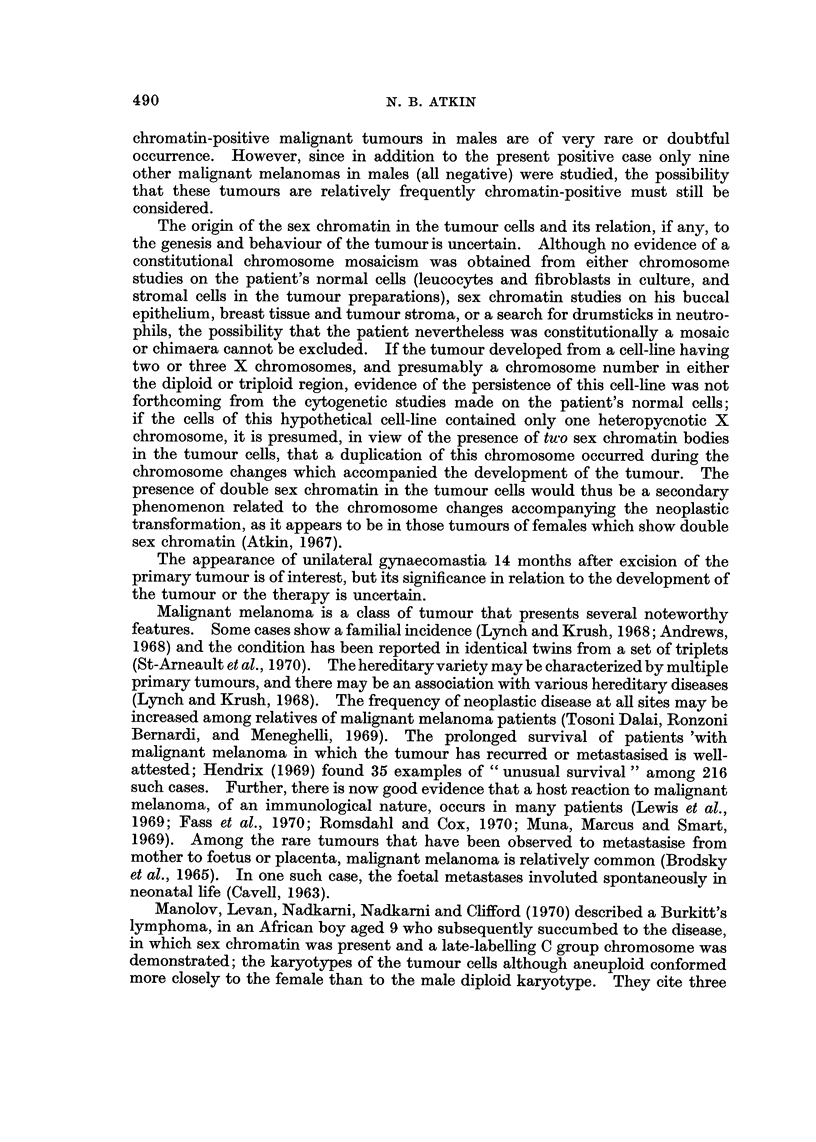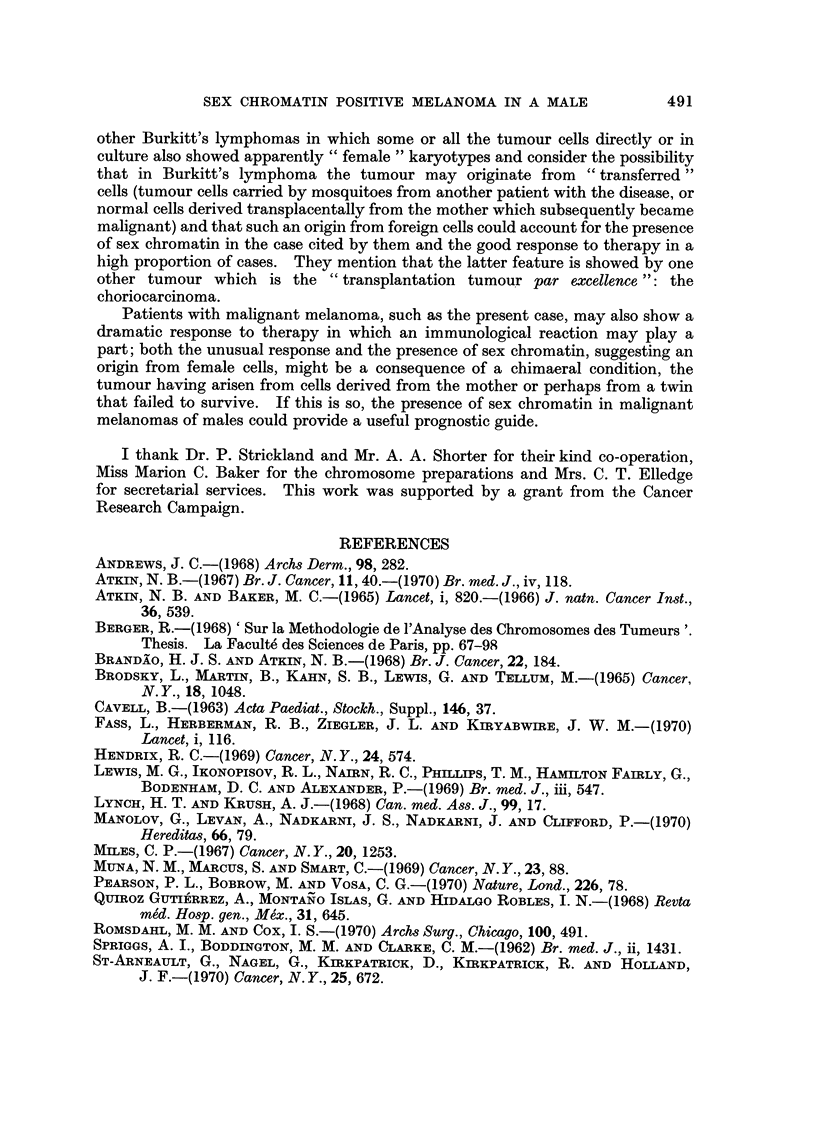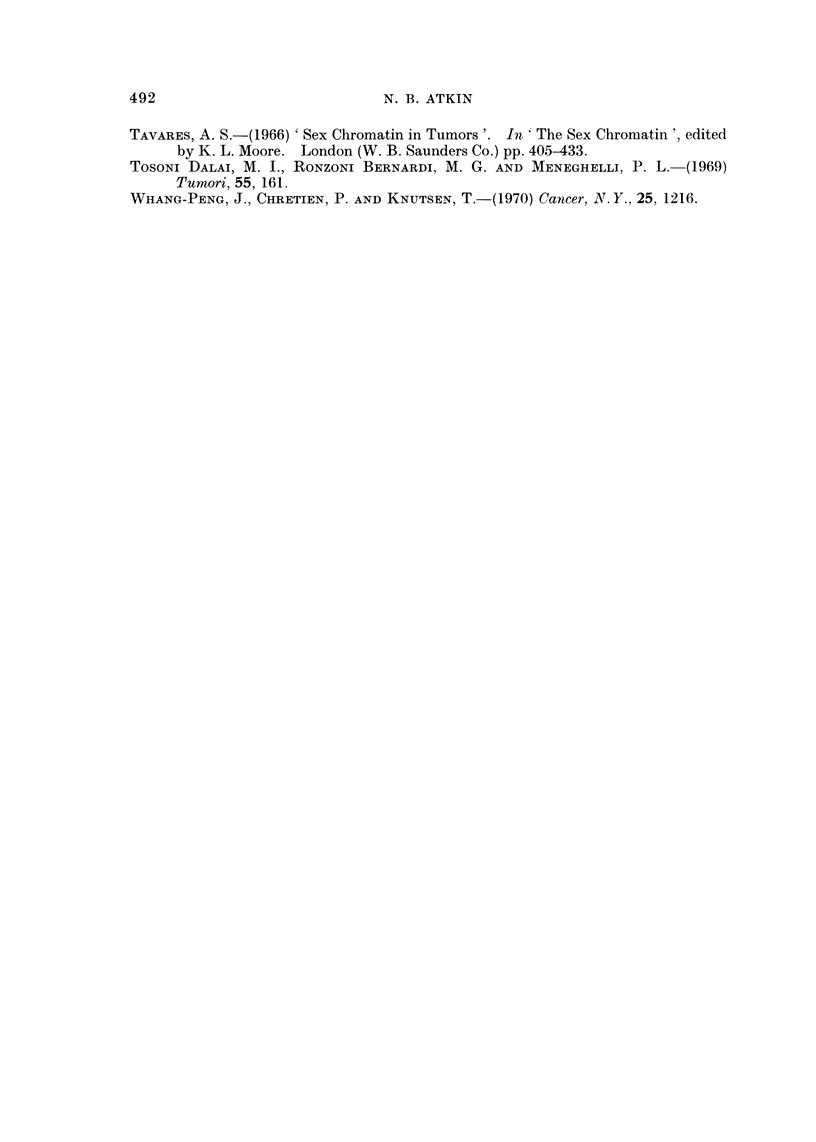# Sex Chromatin Positive Metastatic Melanoma in a Male with a Favourable Prognosis

**DOI:** 10.1038/bjc.1971.61

**Published:** 1971-09

**Authors:** N. B. Atkin

## Abstract

**Images:**


### SEX CHROMATIN POSITIVE METASTATIC MELANOMA IN A MALE WITH A FAVOURABLE PROGNOSIS

N. B. ATKIN

*From the Department of Cancer Research, Mount Vernon Hospital, Northwood, Middlesex*

Received for publication May 25, 1971

**SUMMARY.—The presence of sex chromatin in a metastatic malignant melanoma from a male patient aged 26 who showed no evidence of any constitutional chromosome anomaly is described. A possible association between the apparently " female " origin of the tumour and the good response to therapy is considered.**

TUMOURS of males, apart from teratomas, do not generally show sex chromatin (Tavares, 1966; Atkin, 1967). We found only one chromatin-positive tumour among 311 non-teratomatous malignant tumours studied by a squash technique; this was an oesophageal carcinoma in a patient aged 71 who proved to be an unsuspected case of Klinefelter's syndrome with a 47,XXY karyotype (Atkin and Baker, 1965). The present tumour shows the appearances of sex chromatin in the tumour cells, but studies on the patient's normal cells have revealed no evidence of a constitutional chromosome anomaly. The case is also notable for the good outcome of treatment; the patient is well 4½ years after removal of the primary tumour in spite of the subsequent appearance of metastases.

### CASE REPORT

C.B., aged 26, a clerk, gave a history of a raised pigmented spot of uncertain duration in the right dorsi-lumbar region which had recently bled. The lesion, 1 cm. in diameter, was excised in September 1966; the histological appearances were those of malignant melanoma (Fig. 1). In April 1967, a lump which had appeared over the right 11th rib in the anterior axillary line was excised; on histological examination this proved to be a lymph node replaced by malignant melanoma. Two months later, a block dissection of the right axilla was performed: a number of enlarged lymph nodes were removed, most of which were found to be invaded by amelanotic malignant melanoma although two from the apex of the axilla were free from tumour. The patient was given a postoperative course of telecaesium therapy to the axilla. In October 1967, a nodular indurated area, ? recurrence, ? inflammation, was present in the axilla. A course of tetracycline was then given. At this time a $^{32}P$ uptake test showed some concentration over the nodules which, however, was insufficient to warrant a therapeutic dose of $^{32}P$. A course of melphalan, 5 mg. t.d.s. reduced to 2 mg. t.d.s. after 5 days, was then begun. Four days after commencing the melphalan, enlargement of the right breast appeared; the induration in the axilla also increased and an abscess appeared which was incised. A month later, the axillary condition had greatly improved; a biopsy of the breast was then performed which showed the histological appearances of gynaecomastia (December, 1967).

Throughout the subsequent follow-up period of 38 months, the patient has remained well and free from recurrence. During most of the first 21 months of this period, he was on a daily dose of 2 mg. of melphalan; the melphalan was then discontinued.

### *Family and Previous History*

His father had died of carcinoma of the mouth; his mother, now aged 67, is well. Three brothers and three sisters are alive and well. The patient has had no illnesses of note apart from " congestion of the lungs " on one occasion. He has been married for 3 years, but has no children.

### CYTOGENETIC INVESTIGATIONS

### *Sex chromatin*

Metastatic tumour removed in March and June 1967 showed the presence of sex chromatin; the method of preparation and assessment, using squash preparations stained in aceto-orcein, has been previously described (Atkin, 1967). In approximately 60% of nuclei that were suitable for assessment, *i.e.* free from multiple chromocentres, two Barr bodies were seen (Fig. 2a). After an interval of 2 years, a " blind " reassessment of the original slides, which were coded and examined together with slides of similar age from 16 other malignant cases, six male and 10 female, was made. The sex chromatin findings on all slides were in agreement with those obtained at the original examination.

Histological sections of the primary tumour and the first specimen of metastatic tumour were unsuitable for sex chromatin assessment, but those of the metastatic tumour removed in June 1967 showed chromocentres compatible in appearance with sex chromatin in tumour cells (Fig. 2b).

Sex chromatin was not seen in fibroblasts in the tumour material, epithelial cells in a buccal smear, or epithelial and stromal cells in the breast tissue removed in December 1967. No drumsticks were found among 300 polymorphs in a blood smear.

### *Chromosome studies*

Tumour material removed in March and June 1967 and processed by a direct method (Atkin and Baker, 1966) showed on both occasions a modal number of 68 chromosomes, with similar karyotype changes including two large markers one of

---

### EXPLANATION OF PLATES

FIG. 1.—Histological section of the primary tumour. H. and E. (a) × 6; (b) × 530.

FIG. 2.—Sex chromatin in metastatic tumour (June 1967): (a) orcein squash preparation. Left: × 500; Right: × 1240. (b) histological section. × 2830. Two bodies are usually present.

FIG. 3.—Partial karyotypes of four metaphases from: (a) metastatic tumour (top: April 1967; others: June 1967). Left to right: two different markers, two A1 and five G group chromosomes. Arrows indicate secondary constriction in one of the markers. (b) fibroblast culture: G group chromosomes from diploid metaphases in fibroblast culture. The Y chromosome is on the right of each row.

FIG. 4.—Protruding chromosome arms in histological section of the primary tumour. (a) and (b) metaphases; (c): anaphase; (d): telophase. H. and E. × 1500.

1a

1b

Atkin

2a

Atkin

2b

Atkin

3a

Atkin

3b

Atkin

4a

4b

4c

4d

Atkin which frequently showed a prominent secondary constriction (Fig. 3a). Metaphases, anaphases and telophases in the sections frequently showed chromosomal protrusions suggesting the presence of large marker chromosomes (Brandão and Atkin, 1968); these protrusions were clearly seen in the primary lesion which had a high mitotic index (Fig. 4). The modal number of C group chromosomes was 24. Other karyotypic features most commonly present were: two A1, three A2, two A3, five B, nine D, four E16, four E17/18, eight F and five G group chromosomes. Unfortunately, no attempt was made to identify any late-labelling C group chromosomes autoradiographically. Twelve out of 41 and 23 out of 35 metaphases in the two preparations had apparently normal male diploid karyotypes; presumably these were fibroblasts or other normal cells. Chromosome studies on a blood leucocyte and skin fibroblast culture showed no abnormality; there were no hyperdiploid counts among 43 and 39 metaphases in the respective preparations and analysis of 9 and 15 metaphases respectively showed male diploid karyotypes. A Y chromosome could frequently be identified in the diploid cells, especially in the fibroblast culture (Fig. 3b); the chromosomes of the aneuploid tumour cells were, however, of less good quality, and it was uncertain whether a Y chromosome was present (Fig. 3a).

Application of the quinacrine fluorescence test (Pearson, Bobrow and Vosa, 1970; Atkin, 1970) to tumour and leucocyte-culture material revealed an interphase Y chromatin body in normal cells (fibroblasts and lymphocytes) but none was seen in tumour cells. However, in view of the age of the material when this test was applied it is uncertain whether the failure to observe a fluorescent chromocentre indicates absence of a Y chromosome in the tumour cells.

### *Microspectrophotometric investigations*

Estimation of the Feulgen-DNA content of interphase tumour cells (April and June 1967) showed on each occasion a modal value corresponding to a near-triploid chromosome number.

### *Sex chromatin studies on other malignant tumours of males*

Excluding the present case and the patient with Klinefelter's syndrome already mentioned, sex chromatin has been looked for in squash preparations from 494 malignant tumours of males including those reported in previous studies (Atkin and Baker, 1965; Atkin, 1967). Apart from a thyroid carcinoma which could not be assessed because of the presence of multiple chromocentres in nearly all the tumour cells, all these tumours were sex-chromatin-negative; they include nine malignant melanomas.

### DISCUSSION

Although a number of authors have studied the chromosomes of malignant melanoma cells (Whang-Peng, Chretien and Knutsen, 1970; Miles, 1967; Spriggs, Boddington and Clarke, 1962; Berger, 1968; Quiroz Gutiérrez, Montaño Islas and Hidalgo Robles, 1968), sex chromatin studies appear to be lacking, as do autoradiographic studies with a view to determining whether late-labelling X chromosomes are present. Sex chromatin studies in this laboratory suggest that, excluding testicular teratomas and occasional tumours in subjects with congenital chromosome anomalies involving the presence of additional X chromosomes, chromatin-positive malignant tumours in males are of very rare or doubtful occurrence. However, since in addition to the present positive case only nine other malignant melanomas in males (all negative) were studied, the possibility that these tumours are relatively frequently chromatin-positive must still be considered.

The origin of the sex chromatin in the tumour cells and its relation, if any, to the genesis and behaviour of the tumour is uncertain. Although no evidence of a constitutional chromosome mosaicism was obtained from either chromosome studies on the patient's normal cells (leucocytes and fibroblasts in culture, and stromal cells in the tumour preparations), sex chromatin studies on his buccal epithelium, breast tissue and tumour stroma, or a search for drumsticks in neutrophils, the possibility that the patient nevertheless was constitutionally a mosaic or chimaera cannot be excluded. If the tumour developed from a cell-line having two or three X chromosomes, and presumably a chromosome number in either the diploid or triploid region, evidence of the persistence of this cell-line was not forthcoming from the cytogenetic studies made on the patient's normal cells; if the cells of this hypothetical cell-line contained only one heteropycnotic X chromosome, it is presumed, in view of the presence of *two* sex chromatin bodies in the tumour cells, that a duplication of this chromosome occurred during the chromosome changes which accompanied the development of the tumour. The presence of double sex chromatin in the tumour cells would thus be a secondary phenomenon related to the chromosome changes accompanying the neoplastic transformation, as it appears to be in those tumours of females which show double sex chromatin (Atkin, 1967).

The appearance of unilateral gynaecomastia 14 months after excision of the primary tumour is of interest, but its significance in relation to the development of the tumour or the therapy is uncertain.

Malignant melanoma is a class of tumour that presents several noteworthy features. Some cases show a familial incidence (Lynch and Krush, 1968; Andrews, 1968) and the condition has been reported in identical twins from a set of triplets (St-Arneault *et al.*, 1970). The hereditary variety may be characterized by multiple primary tumours, and there may be an association with various hereditary diseases (Lynch and Krush, 1968). The frequency of neoplastic disease at all sites may be increased among relatives of malignant melanoma patients (Tosoni Dalai, Ronzoni Bernardi, and Meneghelli, 1969). The prolonged survival of patients 'with malignant melanoma in which the tumour has recurred or metastasised is well-attested; Hendrix (1969) found 35 examples of " unusual survival " among 216 such cases. Further, there is now good evidence that a host reaction to malignant melanoma, of an immunological nature, occurs in many patients (Lewis *et al.*, 1969; Fass *et al.*, 1970; Romsdahl and Cox, 1970; Muna, Marcus and Smart, 1969). Among the rare tumours that have been observed to metastasise from mother to foetus or placenta, malignant melanoma is relatively common (Brodsky *et al.*, 1965). In one such case, the foetal metastases involuted spontaneously in neonatal life (Cavell, 1963).

Manolov, Levan, Nadkarni, Nadkarni and Clifford (1970) described a Burkitt's lymphoma, in an African boy aged 9 who subsequently succumbed to the disease, in which sex chromatin was present and a late-labelling C group chromosome was demonstrated; the karyotypes of the tumour cells although aneuploid conformed more closely to the female than to the male diploid karyotype. They cite three other Burkitt's lymphomas in which some or all the tumour cells directly or in culture also showed apparently " female " karyotypes and consider the possibility that in Burkitt's lymphoma the tumour may originate from " transferred " cells (tumour cells carried by mosquitoes from another patient with the disease, or normal cells derived transplacentally from the mother which subsequently became malignant) and that such an origin from foreign cells could account for the presence of sex chromatin in the case cited by them and the good response to therapy in a high proportion of cases. They mention that the latter feature is showed by one other tumour which is the " transplantation tumour *par excellence* ": the choriocarcinoma.

Patients with malignant melanoma, such as the present case, may also show a dramatic response to therapy in which an immunological reaction may play a part; both the unusual response and the presence of sex chromatin, suggesting an origin from female cells, might be a consequence of a chimaeral condition, the tumour having arisen from cells derived from the mother or perhaps from a twin that failed to survive. If this is so, the presence of sex chromatin in malignant melanomas of males could provide a useful prognostic guide.

I thank Dr. P. Strickland and Mr. A. A. Shorter for their kind co-operation, Miss Marion C. Baker for the chromosome preparations and Mrs. C. T. Elledge for secretarial services. This work was supported by a grant from the Cancer Research Campaign.

### REFERENCES

ANDREWS, J. C.—(1968) *Archs Derm.*, **98**, 282.

ATKIN, N. B.—(1967) *Br. J. Cancer*, **11**, 40.—(1970) *Br. med. J.*, iv, 118.

ATKIN, N. B. AND BAKER, M. C.—(1965) *Lancet*, i, 820.—(1966) *J. natn. Cancer Inst.*, **36**, 539.

BERGER, R.—(1968) ' Sur la Methodologie de l'Analyse des Chromosomes des Tumeurs '. Thesis. La Faculté des Sciences de Paris, pp. 67–98

BRANDÃO, H. J. S. AND ATKIN, N. B.—(1968) *Br. J. Cancer*, **22**, 184.

BRODSKY, L., MARTIN, B., KAHN, S. B., LEWIS, G. AND TELLUM, M.—(1965) *Cancer, N.Y.*, **18**, 1048.

CAVELL, B.—(1963) *Acta Paediat., Stockh.*, Suppl., **146**, 37.

FASS, L., HERBERMAN, R. B., ZIEGLER, J. L. AND KIRYABWIRE, J. W. M.—(1970) *Lancet*, i, 116.

HENDRIX, R. C.—(1969) *Cancer, N.Y.*, **24**, 574.

LEWIS, M. G., IKONOPISOV, R. L., NAIRN, R. C., PHILLIPS, T. M., HAMILTON FAIRLY, G., BODENHAM, D. C. AND ALEXANDER, P.—(1969) *Br. med. J.*, iii, 547.

LYNCH, H. T. AND KRUSH, A. J.—(1968) *Can. med. Ass. J.*, **99**, 17.

MANOLOV, G., LEVAN, A., NADKARNI, J. S., NADKARNI, J. AND CLIFFORD, P.—(1970) *Hereditas*, **66**, 79.

MILES, C. P.—(1967) *Cancer, N.Y.*, **20**, 1253.

MUNA, N. M., MARCUS, S. AND SMART, C.—(1969) *Cancer, N.Y.*, **23**, 88.

PEARSON, P. L., BOBROW, M. AND VOSA, C. G.—(1970) *Nature, Lond.*, **226**, 78.

QUIROZ GUTIÉRREZ, A., MONTAÑO ISLAS, G. AND HIDALGO ROBLES, I. N.—(1968) *Revta méd. Hosp. gen., Méx.*, **31**, 645.

ROMSDAHL, M. M. AND COX, I. S.—(1970) *Archs Surg., Chicago*, **100**, 491.

SPRIGGS, A. I., BODDINGTON, M. M. AND CLARKE, C. M.—(1962) *Br. med. J.*, ii, 1431.

ST-ARNEAULT, G., NAGEL, G., KIRKPATRICK, D., KIRKPATRICK, R. AND HOLLAND, J. F.—(1970) *Cancer, N.Y.*, **25**, 672.

TAVARES, A. S.—(1966) ' Sex Chromatin in Tumors '. *In* ' The Sex Chromatin ', edited by K. L. Moore. London (W. B. Saunders Co.) pp. 405–433.
TOSONI DALAI, M. I., RONZONI BERNARDI, M. G. AND MENEGHELLI, P. L.—(1969) *Tumori*, **55**, 161.
WHANG-PENG, J., CHRETIEN, P. AND KNUTSEN, T.—(1970) *Cancer, N.Y.*, **25**, 1216.